# A Constructive Way to Think about Different Hydrothermal Environments for the Origins of Life

**DOI:** 10.3390/life10040036

**Published:** 2020-04-09

**Authors:** Arthur Omran, Matthew Pasek

**Affiliations:** Department of Geosciences 1, University of South Florida, Tampa, FL 33620, USA; mpasek@usf.edu

**Keywords:** submarine hydrothermal vents, hot springs, warm little pond, progenote, chemical evolution, origins of life

## Abstract

The question of where life originated has been contentious for a very long time. Scientists have invoked many environments to address this question. Often, we find ourselves beholden to a location, especially if we think life originated once and then evolved into the myriad forms we now know today. In this brief commentary, we wish to lay out the following understanding: hydrothermal environments are energetically robust locations for the origins and early evolution of life as we know it. Two environments typify hydrothermal conditions, hydrothermal fields on dry land and submarine hydrothermal vents. If life originated only once, then we must choose between these two environments; however, there is no reason to assume life emerged only once. We conclude with the idea that rather than having an “either or” mind set about the origin of life a “yes and” mind set might be a better paradigm with which to problem solve within this field. Finally, we shall discuss further research with regards to both environments.

## 1. Introduction

The questions about the origins of life seem intractable at times. If we try to solve problems with constructive criticism and open mindedness, we may find our current paradigms are what make those questions intractable in the first place. The question of where life originated has been contentious for a very long time. Scientists have invoked many environments to address this question. Often, we find ourselves beholden to a location, especially if we think life originated once and then evolved into the myriad forms we now know today. 

**Figure 1:**
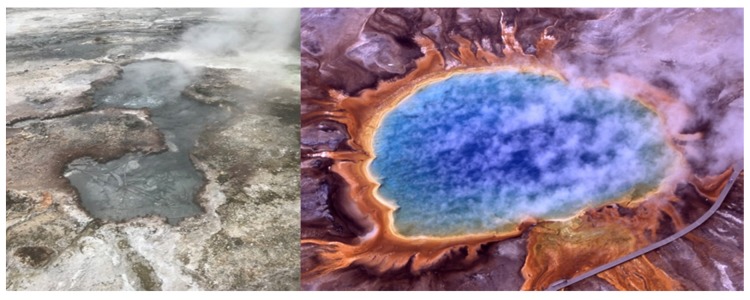
Hydrothermal fields on dry land. (**Left**) Orakei Korako. Upwelling Pool with discharge channel on sinter apron, near Rotorua New Zealand. Photo Credit: Bruce Damer. (**Right**) Grand Prismatic Hot Spring, Mid/Lower Geyser Basin at Yellowstone National Park. Public domain image provided by Jim Peaco at the National Park Service.

**Figure 2:**
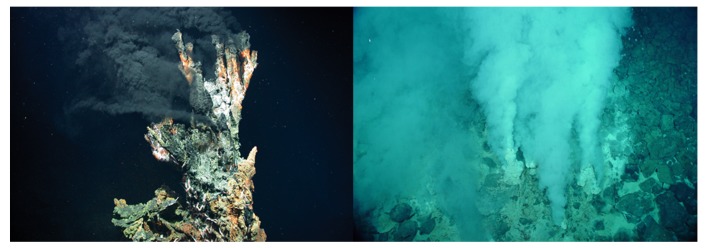
Hydrothermal vent chimneys. (**Left**) the candelabra black smoker hydrothermal vent chimney. Photo credit: Center for Marine Environmental Sciences, University of Bremen. (**Right**) The Champaign vent, a white smoker hydrothermal vent. Public domain image provided by National Oceanic and Atmospheric Administration of the United States.

Has life originated, or evolved from a life-like stage, many times rather than just once? It seems life needs a liquid solvent such as water, must be organic carbon-based, utilizes phosphorus, nitrogen and other elements as nutrients [1], and must have energy as an organizing component to its environment to foster self-assembly. Two environments typify these requirements, hydrothermal fields on dry land (Figure 1) and hydrothermal vents in the sea (Figure 2). If life originated only once, then we must choose between these two environments; however, there is no reason to assume life emerged only once.

In this brief commentary, we wish to lay out the following understanding: hydrothermal environments are energetically robust locations for the origins and early evolution of life as we know it. We will introduce briefly these locations and discuss what makes them so attractive to the study of life’s emergence. We will then conclude with the idea that rather than having an “either or” mind set about the emergence of life a “yes and” mind set might be a better paradigm with which to problem solve within this field.

### 1.1. Hydrothermal Fields on Dry-Land

The idea of a warm pond being the cradle for life to form on an early earth was originally postulated by Charles Darwin [2]. The idea is currently rooted in two strong points, access to biopolymers and natural selection [3,4,5]. An excellent example of hydrothermal fields on dry land would be Yellowstone National Park’s geysers, and the hydrothermal fields near Rotorua New Zealand [6] (Figure 1). The beautiful color banding seen in the pools at Yellowstone are the pigments from chemotrophic and phototrophic microbes living in these extreme environments (Figure 1). These environments on dry land harken back to the idea of a warm pond being the birthplace of life.

Since then, Darwin’s prescient guess has become synonymous with desert ponds and hydrothermal fields on dry land, and many dry land-based pooling systems have been proposed [3,4,5,7,8,9,10,11]. For this modern idea, fresh water accumulated in hydrothermal fields, that underwent natural wet-dry cycling [3,4,5]. During evaporation organic films formed on mineral interfaces, where chemical and physical changes occurred. Products would accumulate in pools when it rained or rehydrated otherwise [5,11]. This wet-dry cycling is one of the most effective methods of polymerizing simple bio-monomers into biopolymers on a lifeless planet [3,4,5,9,10]. These environments would have inputs of water, elements associated with life, and wet-dry cycling; therefore, they should not be rejected from the study of life’s emergence.

Given enough time and wet-dry cycling life may have evolved from a progenote state. A progenote is gel or cell like structure that establishes the linking of genotype to the expression of phenotype. Life may have emerged within these ponds from an interconnected progenote to something resembling a primitive cell [3,4,5]. 

Future work in this field should attempt to address life’s combinatorial emergence, using high powered computation to elucidate the necessary horizontal gene transfer steps for life to emerge. Additionally, the hazards of dry land include impacts from meteors, which were many during the heavy bombardment period, and ultraviolet radiation. Future models and hypotheses should address this weakness and try to set a habitability boundary for the land during the Hadean epoch, if plausible. 

### 1.2. Submarine Hydrothermal Vents

Hydrothermal vents were discovered in 1977 [12] as this was the first ecosystem observed that was devoid of sun light, it was hypothesized that life may have originated in hydrothermal vents [13,14,15]. The logic behind this idea was 3-fold: life needs water, most of which is in the ocean; hydrothermal vents are a source of chemical energy, and populations of chemotrophic microbes live in hydrothermal vents. Additionally, this idea accommodates iron sulfur complexes and their link to metabolism well. Positing the emergence of metabolism and life in a marine environment rich in reactive minerals [14,16,17]. 

There are different types of vent chimneys, black smokers, white smokers, and non-smokers based on different heating sources (Figure 2). These submarine hydrothermal fields all exhibit thriving ecosystems. They form when water meets heated rock deep under the sea [15]. The vent environments are very diverse in mineral composition, pH range and temperature range from one another. Yet, we find life thriving in even the most extreme environments [13]. 

These deep-sea environments have steep energy gradients that potentially drive other chemical processes, processes that would not occur without a free energy source, forward [14,18,19]. These environments have water, energy, gradients, and the necessary elements for life present, so too should they not be neglected from our consideration. Given time, energy, and available gradients, life may have taken hold within the vent membranes, pores, and chambers [14]. Further research in this field should address how polymers form in these environments, particularly in the absence of wet-dry cycles and the immense diluting potential of the surrounding environment.

## 2. Discussion and Conclusions

Hydrothermal environments, both submarine and on dry land, are very appealing to the study of the emergence of life. Geyser systems exhibit wet-dry cycling that is arguably the best method for forming biopolymers before life could do it on its own [5,9,10]. It is a classic idea related to Darwin’s conjecture and ideas like the primordial soup [2]. The older and more established an idea the more work and theory can buttress the idea, making it a solid hypothesis if not more. There is a great body of experimental work showing more than speculation with regards to these ponds and geysers [3,4,5,6]. Given time we may end up referring to this idea as the Theory of Darwin’s Ponds. 

Hydrothermal vents exhibit tremendous energy output unparalleled by any other environment. The vents exert enough energy flowing “downhill” to power any necessary reactions requiring “uphill” energetics [14,18,19]. These vent chimneys exhibit a plethora of gradients that could be useful to prebiotic chemistry and contain pores and membranes where products could accumulate and given time potentially evolve [14]. We have known about the vents’ existence for a shorter period, therefore more work is needed to solidify our understanding of these environments in a prebiotic context. This does not disqualify them from the purview of the study of life’s emergence, rather it should goad us forward with questions from our colleagues, in a constructive way. 

### 2.1. With Regards to Common Ground Between these Environments

What do these environments have in common? They are diverse, exhibiting various pH and temperature ranges. Both environments contain minerals, and fossils of ancient life [20,21]. They have sources of water, carbon, nitrogen, phosphorus etc., supplied by meteors, geologic, or atmospheric inputs, and are plausible on the early Earth. Also, they may both have had access to the type of iron sulfide clusters found within metabolism [7,17]. Importantly we find life thriving in these extreme environments.

These environments may be linked to one another further, with researchers drawing on both as they further their work in a more open-minded fashion [22,23]. Their primary difference being one of elevation from the sea floor, we even find intermediate environments that exhibit aspects of both extremes, specifically we refer to shallow water vent systems. Shallow water systems offer mineral based structures like deep-sea vents. They also exhibit gaseous pockets, which will not form under greater depths and pressures. Additionally, they can be also exposed to UV in clear waters like dry land hydrothermal field systems [22].

One environment or mechanisms’ trash could be another’s treasure. Surface hydrothermal systems may suffer from UV and impacts that may also be the events that initiate the chemistry needed to bring about life in the first place [24,25]. Dilution is a serious problem for underwater hydrothermal systems, requiring concentrators, vent-ocean contact provides sustained electrochemical gradients that may have provided selection pressure for biochemical processes across membranes that may form within hydrothermal pores of submarine vents [26] or products produced elsewhere that end up in the sea. Extreme deep-sea vents may even provide degradation/disposal of waste products that originated in other environments, as a sort of geochemical recycling center for the early Earth [27].

### 2.2. With Regards for the Last Universal Common Ancestor

The last universal common ancestor (LUCA), is the most recent population of organisms from which life on Earth can trace a common descent from. LUCA itself is an idea based on observations via phylogenetics. This concept is like the progenote but assumes a fully established link of phenotype and genotype. The existence of LUCA points many a thinker to an apparent contradiction with the hypothesis of multiple emergences of life; really, one may innocently consider a single origin of life, for explaining a common ancestor.

Current research indicates that the last universal common ancestor’s genetics were probably linked to a hot environment, and therefore LUCA was thermophilic, linking it to a hydrothermal environment [28,29]. Both environments are then prime candidates for early life.

To address the question of having to choose between environments, we cannot be sure life emerged only once. People think of life originating once as a type of creation mythos that is deeply entrenched in our psyches, reinforced by the idea of the LUCA at the bottom of our tree of life. The LUCA probably arose from horizontal gene transfers (HGTs) among many progenote populations in hot environments [30].

Mobile gene elements (MEGs) have many methods of transfer: gene transfer agents, transduction, transformation, conjugation, and membrane vesicle transfer [31]. LUCA may have also contained DNA, indicative of it being evolved from HGT and MEGs and not originally primordial [31]. Indeed, when we investigate genomic data with regards to LUCA or proto “RNA organisms” we find that MEGs were exchanging on an extensive scale, overturning our naive idea that LUCA originated once [31]. Ancient gene families display that HGT occurred in the distant past; moreover, the degree to which HGT was involved in LUCA epoch genetics was dominant and widespread [32,33]. It is with all this in mind that we must admit to ourselves that such a simplistic idea as LUCA originating once, primordially, is on uncertain grounds at best.

With the possibility of life emerging from progenotes it is not unproductive to view these two hydrothermal environments as hosts to the progenotes or supplying materials that could form a progenote. They just have different methods that aid in starting up self-assembly, fields on dry land using wet-dry cycling, and submarine fields using gradients and geothermal energy.

## 3. Further Research

There are many areas for us to study. How a hydrothermal field on dry land makes a cell with a minimal genome is a daunting question. We should look to high power computational modeling for fresh perspectives on this older idea. Modeling of gene transfers within the progenote could yield an idea of what genetic elements are needed to coalesce for a minimally functional cell to take hold from the progenote. Additionally, new hypotheses should incorporate the hazards of said progenote evolving on the land, being exposed to meteor impacts and UV radiation. Once understood, experimental work that tracks genetic elements within putative progenotes could begin.

The great examples of polymer production in dry land-based hydrothermal fields should goad us further to see if we can form polymers in deep-sea hydrothermal field settings, albeit, in a different fashion than wet-dry cycling. A good next step for the hydrothermal vent community to address would be a nonenzymatic process by which catalytic and replicating systems of polymers are produced under plausible prebiotic conditions. This would help to address the “crucial experiment” established by John Platt regarding the origins of life [34]. Addressing this obstacle would allow us to further develop the hydrothermal vent hypothesis in a fashion like, but not limited to, the hot-spring hypothesis [35,36,37]. Once polymers are shown to be produced in this system further work can address the effects of selection on said polymer species.

It is imperative that we as a community, or at least a majority with in our community, do not totally discard the hydrothermal vent hypothesis for any reason, because on worlds with no option for wet-dry cycles, such as the icy moons of Jupiter and Saturn, hydrothermal vents may play a crucial role in establishing the biosphere via other means [38,39,40]. Research into both fields is important for our fundamental understanding of life and may inform us on what frontiers we should investigate next.

### 3.1. The Advantages of Constructive “yes and” Thinking to Our Field

This field is filled with wonder and creativity. Additionally, it is buttressed by assumptions and conjecture due to the nature of what we study. If one does not wish to turtle (or retreat) ideologically inward and we look closely to see what underlies those assumptions, we my find it to be assumptions all the way down. Most work regarding the emergence of life is based on an incomplete understanding of the conditions of the early earth. Additionally, our knowledge of life is limited by our current definitions and understandings of life as we know it. It is with these uncertainties that we should keep an open mind but still try and progress forward. 

The primary advantage of a “yes and” mind set would be to have a less acrimonious dialogue between colleagues. Secondarily, this would lead to less tribalism within the field. Several scientists within the field are coming around to a similar mind set as well [23]. We thought it appropriate to apply this idea to hydrothermal environments both dry land and submarine based, since this division was so very inflammatory at times. Which is amusing to think about, being that the primary difference between the two environments is elevation from the sea floor. Thirdly, we receive a higher degree of utility from constructive criticism, coming from an open-minded well-intentioned colleague. This allows us to accept it more easily and thus make progress more readily.

It may be that life is ubiquitous in the universe and arises via a plethora of mechanisms, therefore an open minded “yes and” mentality would allow us to combine concepts more easily and holistically. This would allow us to pursue multiple origins more readily. For example, one could investigate what happens when materials from a submarine vent are moved to a dry land environment or vice versa. Could waste products formed in one place be degraded by another as a form of recycling between environments? Finally, could interconnected environments play a role in selective pressure and its fluctuation across progenotes promoting HGT?

### 3.2. Something Else Darwin Once Said

If we can agree that life could have arisen more than one time, then there is no need to for it to arise in only one place, in only one way. Charles Darwin speculated that life may have emerged once or a few times.

“There is grandeur in this view of life, with its several powers, having been originally breathed into a few forms or into one; and that, whilst this planet has gone cycling on according to the fixed law of gravity, from so simple a beginning endless forms most beautiful and most wonderful have been, and are being, evolved.”—Charles Darwin, The Origin of Species.

Grandeur in several powers into few forms, we get simple beginnings that continuously evolve. This is incredibly useful and need not be limited to one environment or one paradigm, life could emerge in many ways, in many places. It does not have to be “either only this paradigm is right, or another is” it can be “yes, this paradigm is plausible and so others may be as well”.
